# Analysis of Age-Related Global DNA Methylation in Chicken

**DOI:** 10.1007/s10528-013-9586-9

**Published:** 2013-04-04

**Authors:** Magdalena Gryzinska, Ewa Blaszczak, Aneta Strachecka, Grazyna Jezewska-Witkowska

**Affiliations:** 1 Department of Biological Basis of Animal Production, Faculty of Biology and Animal Breeding, University of Life Sciences in Lubin, Lublin, Poland; 2 CNRS, UMR 6290, Institut de Génétique et Développement de Rennes, 5043 Rennes, France; 3 Faculté de Médecine, Université de Rennes 1, UEB, IFR 140, 5043 Rennes, France

**Keywords:** Epigenetics, Global DNA methylation, Aging, Chicken, Polbar

## Abstract

DNA methylation is an epigenetic modification that plays an important role in the normal development and function of organisms. The level of DNA methylation is species-, tissue-, and organelle-specific, and the methylation pattern is determined during embryogenesis. DNA methylation has also been correlated with age. The aim of this study was to determine the global DNA methylation levels and their correlation with age in the chicken, using a Polish autosexing chicken breed, Polbar. A quantitative technique based on an immunoenzymatic assay was used for global DNA methylation analysis. The results show increased global DNA methylation levels with older Polbar embryos. Global DNA methylation levels decrease with the age of hens in the postembryonic stage. This study expands the current knowledge of the Polbar epigenome and the general knowledge of the function of epigenetic mechanisms in birds.

## Introduction

The term “epigenetics” was introduced by Waddington (1942) and defined as “the branch of biology which studies the causal interactions between genes and their products, which bring the phenotype into being.” Since then, various definitions have been proposed and various biological phenomena have been placed in the category of epigenetics. In current understanding, epigenetics is the study of heritable changes in gene expression and cellular phenotype that are not associated with changes in the DNA sequence, and it is “a bridge between genotype and phenotype” (Goldberg et al. 2007). DNA methylation is an example of epigenetic modification because of its heritability during mitotic division and its ability to influence gene expression without changes in the DNA sequence. It is one of the most frequent biochemical processes in eukaryotic genomes (Bird 2002; Sulewska et al. 2007). In this process, the methyl group (–CH_3_) is covalently attached mainly to the pyrimidine ring of cytosine, creating 5-methylcytosine. The attachment is catalyzed by DNA methyltransferases, which use S-adenosylmethionine as the donor of the methyl group (SAM–CH_3_). This mechanism was proposed by Wu and Santi (1987; Zhu 2006).

DNA methylation is a species-, tissue-, and organelle-specific process that has been shown to play a crucial role in the normal development and function of organisms. Aberrations in global DNA methylation patterns have been associated with aging, carcinogenesis, and some inherited disorders (Laird 1997; Baylin and Herman 2000; Robertson and Wolffe 2000; Arney and Fisher 2004). Over the years, differences in genome methylation profiles have been observed, depending on the age and sex of an organism. Several studies investigating age-dependent DNA methylation have been carried out on mammals (Romanov and Vanyushin 1981; Dunican et al. 2008; Bocklandt et al. 2011). In mammalian cells the highest percentage of DNA methylation occurs during the S-phase of the cell cycle, and the DNA methylation pattern changes during normal organism development. A dramatic decrease in DNA methylation levels, called global demethylation, takes place in the early embryonic stage of development. After implantation, the majority of the male mammalian genome remains methylated, whereas the female genome undergoes passive demethylation. Tissue-specific genes during gastrulation are also demethylated. The next step of de novo methylation occurs during gametogenesis and is sex dependent (Lee et al. 2004; Allegrucci et al. 2005). In the mouse and other model organisms, such as *Danio rerio* and *Xenopus laevis*, a decrease in the expression of DNA methyltransferase 1 leads to a lethal phenotype (Damelin and Biestor 2007).

The correlation between the age of an organism and DNA methylation levels was noted by Vanyushin et al. (1969), who reported a decrease in DNA methylation with the age of salmon. Numerous studies confirmed these findings by investigating rat, mouse, and bovine organs. For example, in rats a notable loss of genomic methylation was observed in the brain, heart, spleen, and liver in older specimens (reviewed in Pogribny and Vanyushin 2009). In the embryonic development of mice, a decrease in methylation status of about 30% was observed as embryos aged (Bird 2002). Holliday (1985) first hypothesized that changes in DNA methylation are significant in the aging process. Since then, a considerable amount of literature has been published on DNA methylation and aging (Wilson et al. 1987; Ono et al. 1993; Ahuja and Issa 2000; Richardson 2002; Fraga and Esteller 2007). In humans, Boks et al. (2009) observed a correlation among DNA methylation pattern, age, and sex.

Today little is known about DNA methylation in birds (Li et al. 2011). Birds and mammals shared a common ancestor around 300 million years ago. The chicken (*Gallus gallus*) is an important animal model for studying vertebrate development, as it “bridges an evolutionary gap” (Burt and Pourquie 2003). Recent evidence suggests that the DNA methylation pattern in the chicken is similar to that in mammals (Li et al. 2011) and that methylation patterns vary among chicken breeds and among various tissues (Xu et al. 2007). Nätt et al. (2012) also suggested that patterns of DNA methylation are inherited in chickens despite differences in DNA sequence, which may play a role in domestication-related phenotypic changes.

**Fig. 1:**
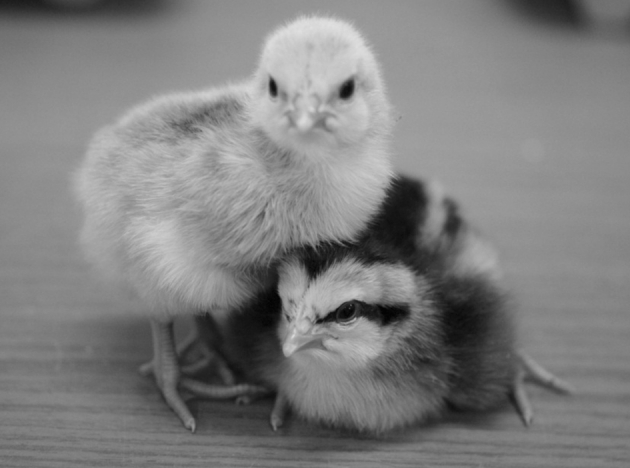
Polbar chicks one day after hatching. The male (*standing*) has creamy-yellow feathers; the female has dark feathers and a characteristic black streak traversing the eye

Our study investigated changes in global DNA methylation levels during Polbar chicken development. The Polbar chicken is a Polish autosexing breed, so it is possible to distinguish the sex of the chicks immediately after they hatch: males have creamy-yellow feathers, and females are dark, with a characteristic black streak that traverses the eye (Fig. 1). Created by Prof. Laura Kaufman between 1946 and 1954 by crossing Greenleg Partridge females with Plymouth Rock males, the breed was named Polbar because the gene *bar* was introduced into the native Polish breed (Kaufman 1963; Lorkiewicz 1976; Gryzinska and Niespodziewanski 2009). The Polbar chicken breed, reared originally for scientific purposes, belongs to a conservative flock, but it is also a very good commercial hen breed (Gryzinska and Niespodziewanski 2009).

The aim of this study was to determine the age-related global DNA methylation pattern in the embryonic and postembryonic period of Polbar development. At present, no data have been published on global DNA methylation levels in a chicken model, using a quantitative method of analysis based on an immunoenzymatic assay. Moreover, this is the first study to report the global DNA methylation pattern of an autosexing chicken breed.

## Materials and Methods

The materials for this study were obtained from Polbar chickens maintained in the Laura Kaufman Research Station at the University of Life Sciences in Lublin, Poland. The Polbar chicken was selected because it is a native Polish breed maintained as a conservative flock, and the university’s flock is the only population of the breed that is being preserved. The samples for this study were taken from Polbar embryos during days 6, 12, and 18 of incubation; from the blood of chicks one day after hatch; and from the blood of 32-week-old hens. All procedures were approved by the Local Ethics Committee on Animal Research in Lublin (No. 8/12 from 15.03.2011). All reagents were of molecular biology or analytical grade.

### DNA Isolation and Quantification

DNA was extracted from Polbar embryos at 6, 12, and 18 days old using a DNeasy Tissue Kit (Qiagen), according to the manufacturer’s protocol. DNA was isolated from 20 mg of tissue cut into small pieces, following the supplier’s recommendation, to enable more efficient lysis. DNA from the blood of one-day-old chicks and 32-week-old hens was isolated using a QIAamp DNA Blood Mini Kit (Qiagen), according to the manufacturer’s protocol, modified to reduce the total starting blood volume to 50 μl, with an appropriate pipetting of samples. Briefly, 50 μl blood, 20 μl proteinase K, and 350 μl lysis buffer AL were added to the microcentrifuge tube. Samples were mixed by pipetting until a beige foam was obtained. Samples were incubated at 56°C for 10 min, and the rest of the procedure was carried out as stated in the protocol. DNA was eluted in 200 μl elution buffer AE supplied by the kit’s manufacturer. For quantification analysis, 50 μl of each DNA sample was taken. DNA quantification was performed spectrophotometrically by measuring the absorbance at 260 and 280 nm using a BioPhotometer (Eppendorf). DNA samples with an A_260_/A_280_ ratio of 1.7–2.0 were used for methylation analysis. DNA quality was assessed by electrophoresis at 120 V for 40 min, using a 1% agarose gel stained with ethidium bromide. DNA was visualized using a Syngene BTX 26M transilluminator.

### Global DNA Methylation Analysis

The global DNA methylation analysis was performed using a commercially available methylated DNA quantification kit (MDQ1, Imprint, Sigma-Aldrich) to detect relative levels of methylated DNA, based on the ELISA principle. A 96-well plate format was used. The DNA samples were diluted in binding solution, and the amount of DNA used was calculated to give a final concentration of 150 ng/μl. DNA binding was achieved by incubating 30 μl diluted DNA at 37°C for 1 h. Block solution was added, and samples were incubated again at 37°C for 30 min. Methylated DNA was then captured using diluted capture antibodies and detected by binding to the previously diluted detection antibodies. As stated by the kit manufacturer, after the addition of a developing solution to the wells, a colorimetric reaction occurs (the solution turns blue). Therefore, color changes were monitored, both after the addition of the developing solution and 10 min after incubation, when the stop solution was added (the solution turned yellow). Both a blank control and a methylated control (positive control) were analyzed together with the DNA samples. The absorbance was measured at 450 nm, because the amount of methylated DNA present in each sample was proportional to the measured absorbance. For each developmental time point, six samples, each derived from a different chicken, were analyzed. The absorbance measurements were performed in duplicate, each using the same amount of DNA derived from the same organism (Table 1). The measurements for the methylated control and the blank sample were also performed in duplicate. The replicates were averaged, and the mean value was used for further analyses. Global DNA methylation levels are percentages relative to the methylated control provided and were calculated using the following equation:

$$ \frac{{{\text{A}}_{450} {\text{S}} - {\text{A}}_{450} {\text{B}}}}{{{\text{A}}_{ 4 5 0} {\text{MC}} - {\text{A}}_{450} {\text{B}}}} \times 100\% $$

where A_450_S is the average absorbance of the sample, A_450_B the average absorbance of the blank, and A_450_MC the average absorbance of the methylated control.

**Table 1:** DNA and binding solution amounts used to obtain a DNA concentration of 150 ng/μl in each sample

Sample	Day 6		Day 12		Day 18		Day 1 after hatch		32 weeks	
	DNA (μl)	Binding solution (μl)	DNA (μl)	Binding solution (μl)	DNA (μl)	Binding solution (μl)	DNA (μl)	Binding solution (μl)	DNA (μl)	Binding solution (μl)
1	7.89	22.11	5.36	24.64	4.41	25.59	3.57	26.43	3.57	26.43
2	15.00	15.00	10.00	20.00	4.83	25.17	8.33	21.67	4.29	25.71
3	6.00	24.00	6.82	23.18	3.26	26.74	6.25	23.75	3.67	26.33
4	6.00	24.00	5.17	24.83	3.06	26.94	7.50	22.50	4.69	25.31
5	5.17	24.83	5.56	24.44	3.95	26.05	3.00	27.00	3.85	26.15
6	5.36	24.64	5.77	24.23	4.29	25.71	4.29	25.71	2.63	27.37

The analyses were performed separately for the two study periods because material collected during the embryonic period (days 6, 12, and 18) derived from solid tissues and the material collected during the postembryonic period (day 1 after hatch, and 32 weeks) derived from blood.

### Statistical Analysis

Statistical analysis of the data was performed using Statistica version 10 (StatSoft) and Microsoft Office Excel 2007. First we applied the Shapiro–Wilk normality test to determine whether parametric or nonparametric statistical tests should be used for further analysis. Then Levene’s test was used to assess equality of variances and to determine whether ANOVA could be used for further analysis. Following confirmation with these tests, the Kruskal–Wallis nonparametric test was used to evaluate differences in global DNA methylation levels within samples derived from different days of the embryonic period. To evaluate the data from the postembryonic period, one-way ANOVA was used. *P* values of 0.05 or lower were considered significant. For both study periods, the coefficient of variation of the assay was calculated as (SD/mean) × 100%. Pearson’s correlation was used to assess the correlation between continuous variables.

## Results

**Fig. 2:**
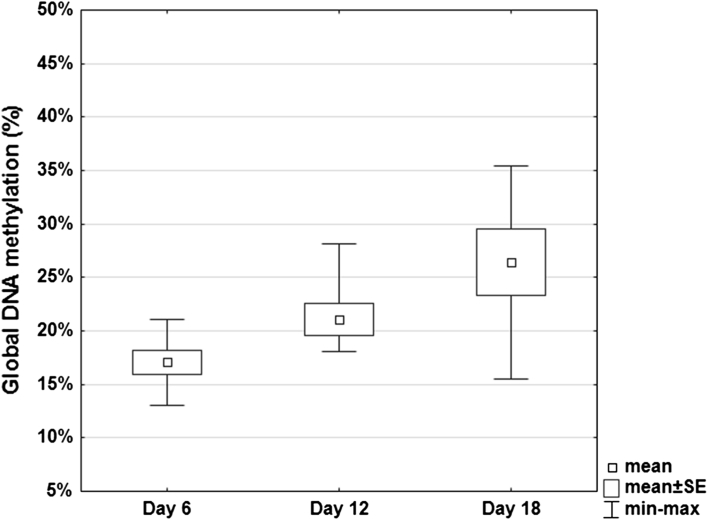
Global DNA methylation pattern of 6-, 12-, and 18-day-old Polbar embryos (the embryonic period) relative to the methylated control. *Box* indicates mean ± standard error for each day, *small square* within the box indicates the mean for that day, and the *horizontal bars* indicate maximum and minimum values; *n* = 6 for each age

Global DNA methylation levels were estimated as percentages of methylated cytosines in the samples, relative to the methylated positive control. The global DNA methylation levels of the embryonic Polbar chickens increased significantly (*p* = 0.028), from 17.09 to 26.47% between days 6 and 18 of incubation (Fig. 2). The Shapiro–Wilk test and Levene’s test were used to check for normal distribution and homogeneity of variance. The Shapiro–Wilk test confirmed that the samples came from a normally distributed population. Levene’s test, however, yielded a *p* value of 0.022 (heterogeneity of variance), excluding the use of ANOVA for further analysis for this particular study period. Therefore, the Kruskal–Wallis test was used to assess statistical significance. An increase in methylation levels was also observed between day 6 (mean = 17.09%) and day 12 (mean = 21.08%) of embryo incubation, but no significant difference was found between days 6 and 12 (*p* = 0.31) or between days 12 and 18 (*p* = 0.99) of development. The coefficient of variation showed variability among the samples for day 6 (16.26%), day 12 (17.41%), and day 18 (29.08%) of the embryonic period. Pearson’s product-moment correlation (*r*) was used to determine the relationship between methylation levels on days 6 and 18 of embryonic incubation, and the resulting *r* value of 0.96 indicated that the two variables increased together.

**Fig. 3:**
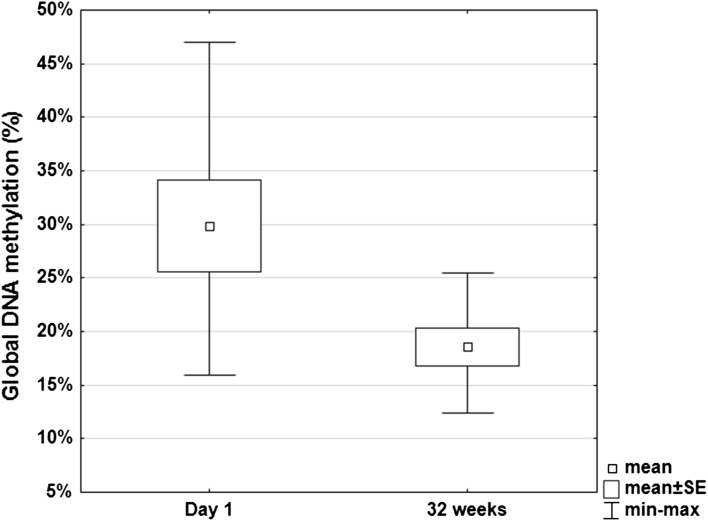
Global DNA methylation pattern of one-day-old Polbar chicks and 32-week-old hens (the postembryonic period) relative to the methylated control. *Box* indicates mean ± standard error for each day, *small square* within the box indicates mean, and the *horizontal bars* indicate maximum and minimum values; *n* = 6 for each age

During the postembryonic period, global DNA methylation levels decreased significantly (*p* = 0.036), from 29.89% for the one-day-old chicks to 18.56% for the 32-week-old hens (Fig. 3). Statistical significance was confirmed by one-way ANOVA, which was an appropriate statistical model because the Shapiro–Wilk test confirmed normal distribution and Levene’s test confirmed the assumption of homogeneity of variance (*p* = 0.172). The coefficient of variation was 35.14% for the one-day-old chicks and 23.65% for the 32-week-old hens. The coefficient of variation for total assay variability was greater in the postembryonic period (40.00%) than in the embryonic period (29.04%). Pearson’s correlation coefficient confirmed a high correlation between variables in the postembryonic period (*r* = −1).

## Discussion

The aim of this study was to assess the correlation of global DNA methylation patterns and age of Polbar chickens in both embryonic and postembryonic stages of development. To our knowledge, this is the first study to report differences in age-dependent global DNA methylation levels in a chicken model, using a commercial kit based on an immunoenzymatic assay. Prior studies, in particular those on mammals, have noted the dependence of methylation on age of the organism (Dunican et al. 2008; Bocklandt et al. 2011). A study conducted by Li et al. (2011) on DNA methylation patterns in the chicken showed that the chicken DNA methylation profile is analogous to that of mammals and plants (Boks et al. 2009). Little is known regarding the correlation between DNA methylation and age in birds. We hypothesized that the global DNA methylation pattern of Polbar chickens changes with their age, and the results of this study confirm this hypothesis.

Epigenetic modifications pique the interest of many scientists because of the important role they play in the physiology and pathology of the cell (Allegrucci et al. 2005). DNA methylation is one of the epigenetic modifications that take part in the development and function of an organism. It has also been associated with carcinogenesis and the process of aging. Methylation patterns have been shown to be specific to tissues, organelles, and species and to differ with age. Age-related differences in methylation patterns were observed for the first time in 1969 by Vanyushin et al. (reviewed in Vanyushin 2005), whose data showed a decrease in the DNA methylation of humpback salmon during ontogenesis. Other authors had similar findings in studies based on rats, mice, and bovine organs. Some studies, however, have shown an increase in DNA methylation in a particular gene or tissue as an organism ages (Kwabi-Addo et al. 2007).

Our results indicate that global DNA methylation patterns change as a chicken ages. It has been shown that DNA methylation is a tissue-specific process; overall methylation variation in different tissues as well as in different chicken breeds was reported by Xu et al. (2007). Thus, there is a possibility that the global DNA methylation pattern in Polbar chickens is driven by the type and composition of tissues. Therefore, because our study was based on tissue samples for the embryonic period and on blood samples for the postembryonic period, we analyzed our data from the two periods separately in order to exclude tissue as a factor, leaving age as the only analyzed factor. Nevertheless, it must be noted that tissue composition may have had a slight influence on the methylation levels we observed.

In our study, analysis of the embryonic period showed a significant increase in global DNA methylation between days 6 and 18 of embryo development. The differences were not statistically significant, however, between days 6 and 12 or between days 12 and 18. This finding might be related to a limited sample size, the chosen days of development, or a measurement error. We tried to minimize measurement error during the study by collecting samples from embryos incubated in the same incubator and from adult chickens that had been bred and raised in the same environment. Moreover, we performed the measurements in duplicate on the same day and using the same equipment. Although the data from individual samples varied, the results show that variation during the analyzed embryonic period can be characterized as low.

In contrast to the increase in methylation during the embryonic stage of development, we observed a significant decrease in methylation levels during the postembryonic stage, as measured in the one-day-old chicks and the 32-week-old hens. Here the variability between the samples was greater than that in the embryonic samples and can be characterized as average variability. It could be hypothesized that the environment influences differences in methylation profiles; therefore, we tried to eliminate some of the external factors that could contribute to error. The most differentiating factor was the age of the organism. Sampling a greater number of individual organisms in future studies, as well as taking samples at more points during chicken development, would certainly reduce the potential for error.

Our findings suggest that in general the global DNA methylation level of Polbar chickens is age dependent. The methylation pattern differs between stages of development, increasing during the embryonic stage and decreasing during the postembryonic stage. More research needs to be conducted to confirm that the age of chickens is associated with an increase and/or decrease of their DNA methylation levels. In future investigations, it might be advantageous to examine other periods in chicken development to confirm our general findings. The present study broadens the knowledge of the Polbar epigenome and of epigenetic mechanisms in birds. It also shows that the Imprint Methylated DNA Quantification Kit is a quick and easy method for detecting global DNA methylation levels. The correlation between DNA methylation and organism age is an important subject for study, and the unique autosexing Polbar chicken breed is a suitable model organism. This study was not specifically designed to evaluate methylation patterns according to the sex of chickens, because of a lack of biological replicates. Our findings do, however, provide insights for future research on DNA methylation in the Polbar and other chicken breeds, including research on whether methylation patterns may be sex-related. Further research in this field could therefore explore the methylation of genes responsible for sex-linked barring phenotypes.
